# First record of the predatory stink bug species *Picromerusgriseus* (Dallas) (Hemiptera, Heteroptera, Pentatomidae, Asopinae) in Japan, with an illustrated key to the Japanese species of the genus *Picromerus* Amyot & Serville

**DOI:** 10.3897/BDJ.11.e105293

**Published:** 2023-05-17

**Authors:** Jun Souma, Akihiro Utagawa, Tadashi Ishikawa

**Affiliations:** 1 Shirakami Research Center for Environmental Sciences, Faculty of Agriculture and Life Science, Hirosaki University, Hirosaki-shi, Aomori, Japan Shirakami Research Center for Environmental Sciences, Faculty of Agriculture and Life Science, Hirosaki University Hirosaki-shi, Aomori Japan; 2 Omata-machi, Ashikaga-shi, Tochigi, Japan Omata-machi Ashikaga-shi, Tochigi Japan; 3 Laboratory of Entomology, Faculty of Agriculture, Tokyo University of Agriculture, Atsugi-shi, Kanagawa, Japan Laboratory of Entomology, Faculty of Agriculture, Tokyo University of Agriculture Atsugi-shi, Kanagawa Japan

**Keywords:** Heteroptera, Pentatomidae, Asopinae, *Picromerusgriseus*, stink bug, new record, illustrated key, Japan, Ryukyu Islands, Ishigaki Island, Oriental Region

## Abstract

**Background:**

The predatory stink bug genus *Picromerus* Amyot & Serville, 1843 (Hemiptera, Heteroptera, Pentatomidae, Asopinae) comprises 11 species found in the Northern Hemisphere. In Japan, two species have been recorded to date. However, an easy-to-understand identification method, such as an illustrated key, is lacking. Currently, *Picromerusgriseus* (Dallas, 1851) has been recorded in Bangladesh, Bhutan, China, Indonesia, Myanmar, Pakistan and Taiwan, but not in Japan.

**New information:**

*Picromerusgriseus* was recorded in Japan for the first time, based on a single individual collected from grasslands around the fields of Ishigaki Island of the Ryukyu Islands, which belong to the Oriental Region. This discovery represents the easternmost record of the species. An illustrated key to the species of *Picromerus* occurring in Japan is also provided.

## Introduction

The stink bug subfamily Asopinae Spinola, 1850 comprises 303 species in 63 genera worldwide and all species for which their biology is known are predacious (Schuh and Weirauch 2020). The following twelve species in eight genera are known in Japan: *Andrallusspinidens* (Fabricius, 1787); *Armacustos* (Fabricius, 1794); *Dinorhynchusdybowski* Jakovlev, 1876; *Eocantheconafurcellata* (Wolff, 1811); *E.japonica* (Esaki & Ishihara, 1950); *E.kyushuensis* (Esaki & Ishihara, 1950); *E.shikokuensis* (Esaki & Ishihara, 1950); *Picromerusbidens* (Linnaeus, 1758); *P.lewisi* Scott, 1874; *Pinthaeussanguinipes* (Fabricius, 1781); *Rhacognathuscorniger* Hsiao & Cheng, 1977; and *Zicronacaerulea* (Linnaeus, 1758) (Ishikawa 2016). Two Japanese species, *A.spinidens* and *E.furcellata*, are expected to have some success in effectively utilising indigenous natural enemies through conservation (Rural Culture Association 2016).

The genus *Picromerus* Amyot & Serville, 1843 (Asopinae) comprises 11 species from the Northern Hemisphere: *P.bidens*; *P.brachypterus* Ahmad & Önder, 1990; *P.conformis* Herrich-Schäffer, 1841; *P.elevatus* Zhao, Liu & Bu, 2013; *P.fasciaticeps* Zheng & Liu, 1987; *P.griseus* (Dallas, 1851); *P.lewisi*; *P.nigridens* (Fabricius, 1803); *P.orientalis* Rishi & Abbasi, 1973; *P.pseudobidens* Ahmad & Önder, 1990; and *P.viridipunctatus* Yang, 1934 (Thomas 1994, Rider 2006, Zhao et al. 2013). In Japan, two species, *P.bidens* and *P.lewisi*, were only recorded from Japan proper and its surrounding islands, which belong to the Palearctic Region (Ishikawa 2016), while no species have been recorded from the Ryukyu Islands, whose central and southern parts belong to the Oriental Region. These two species are so similar that it is difficult to distinguish them at first glance. Although some field guides and pictorial books dealing with Japanese Asopinae have been published (e.g. Yasunaga et al. (1993), Takai and Ishikawa (2012)), no identification key illustrating the diagnostic characteristics of the two species, based on the Japanese populations, has been provided for easier and more accurate identification. Therefore, field surveys on the Ryukyu Islands and publication of an illustrated key for Japanese *Picromerus* are needed to elucidate Asopinae in Japan and make this genus known to the public.

In autumn, 2022, the second author collected a single individual of an indeterminate species of *Picromerus* from the grasslands around the fields of Ishigaki Island of the Ryukyu Islands (Oriental Region). After the first author examined its morphological characteristics, we concluded that it belonged to *P.griseus*, which is currently known to occur in Bangladesh, Bhutan, China, Indonesia, Myanmar, Pakistan and Taiwan (Thomas 1994, Rider 2006, Zhao et al. 2013, Zheng and Lin 2013). Herein, we report *P.griseus* in Japan for the first time, representing the easternmost occurrence of this species. In addition, we provide an illustrated key for all three Japanese species of *Picromerus*.

## Materials and methods

Morphological characteristics of dried specimens were observed using a stereoscopic microscope (SZ60; Olympus, Tokyo, Japan). To examine the genital characteristics, the male terminalia were removed from the body after softening the specimens in hot water. The removed genital capsule was immersed in hot 15% potassium hydroxide (KOH) solution for 5 min. For further observations, parameres were removed from the genital capsule soaked in 99% ethanol. Male genitalia were preserved in small polyethylene vials containing a 50% glycerine and 50% water solution. A polyethylene vial was mounted on the pin with the specimens. The specimens were photographed using a digital microscope (Dino-Lite Premier M; Opto Science, Tokyo, Japan) and a compact digital camera (Tough TG-6; Olympus) and image stacks were processed using Adobe Photoshop 2021 ver. 22.5.1 (Adobe Inc., CA, USA) when using the digital microscope. Measurements were obtained using a stereoscopic microscope equipped with an ocular grid and a digital microscope. Morphological terms were assigned as described by Tsai et al. (2011).

The single specimen of *Picromerusgriseus* examined in the present study was deposited at the Laboratory of Entomology, Faculty of Agriculture, Tokyo University of Agriculture, Kanagawa, Japan (TUA). Specimens of the Japanese species of *Picromerus* that were used for creating the identification key and for comparison with *P.griseus* were deposited in the Entomological Laboratory, Faculty of Agriculture, Kyushu University, Fukuoka, Japan (ELKU) and TUA.

## Taxon treatments

### Picromerus griseus

(Dallas, 1851)

77EEBAC0-304E-5F94-8CDF-CE8E4A7B3777

*Cantheconagrisea* Dallas, 1851 - Dallas (1851): 92, new species and description.
*Picromerusobtusus* Walker, 1867 - Walker (1867): 133, new species and description; Schouteden (1907): 25, synonymised with *Picromerusgriseus*.
*Picromerusnigrivitta* Walker, 1867 - Walker (1867): 133, new species and description; Distant (1900): 58, synonymised with *Picromerusobtusus*.
*Picromerussundanus* Breddin, 1902 - Breddin (1902): 96, new species and description; Thomas (1994): 192, synonymised with *Picromerusgriseus*. Gaedike (1971): 100, designation of lectotype.
*Picromerusgriseus* Schouteden, 1907 - Schouteden (1907): 25, new combination; Thomas (1994): 192, catalogue and distribution; Rider (2006): 243, catalogue and distribution; Zhao et al. (2013): 73, diagnosis, figure, and distribution; Zheng and Lin (2013): 145, figure and distribution.

#### Materials

- **Type status:** Other material. **Occurrence:** recordedBy: Akihiro Utagawa; individualCount: 1; sex: male; lifeStage: adult; occurrenceID: B9C40A18-594B-59E7-BCA1-4AB46599AC3E; **Taxon:** scientificName: *Picromerusgriseus* (Dallas, 1851); namePublishedIn: 1851; kingdom: Animalia; phylum: Arthropoda; class: Insecta; order: Hemiptera; family: Pentatomidae; genus: Picromerus; specificEpithet: *griseus*; scientificNameAuthorship: Dallas; **Location:** islandGroup: Ryukyu Islands; island: Ishigaki Island; country: Japan; countryCode: Okinawa; municipality: Ishigaki-shi; locality: Sakieda; decimalLatitude: 24.438250; decimalLongitude: 124.102167; geodeticDatum: WGS84; **Identification:** identifiedBy: Jun Souma; dateIdentified: 2023; **Event:** samplingProtocol: none specified; eventDate: 08-11-2022; **Record Level:** institutionCode: TUA; basisOfRecord: PreservedSpecimen

#### Diagnosis

*Picromerusgriseus* can be distinguished from other species of the genus using a combination of the following characteristics: head, pronotum, scutellum and femora uniformly brown (Fig. 1a, b); humeral angle of pronotum strongly protruding laterad, acute at apex, posteriorly with a distinct subapical prominence (Fig. 2a); posterior margin of genital capsule weakly curved inwards in middle part (Fig. 4a); and paramere weakly curved inwards in apical part in dorsal and caudal views, distinctly concave along inner margin in dorsolateral view (Figs 3a, 5a, 6a).

#### Distribution

Japan (Ryukyu Islands: Ishigaki Island), Bangladesh, Bhutan, China, Indonesia, Myanmar, Pakistan, Taiwan (Thomas 1994, Rider 2006, Zhao et al. 2013, Zheng and Lin 2013, present study).

The discovery of *Picromerusgriseus* from Japan represents the easternmost record of the species.

#### Biology

*Picromerusgriseus* was collected from grasslands surrounding fields in Japan. In Japan, adult*s* are collected in November; however, the nymphs are unknown.

#### Taxon discussion

The specimen recorded above (Figs 1a, b, 2a, 3a, 4a, 5a, 6a) matched the photographs and descriptions (Dallas 1851, Zhao et al. 2013) of *Picromerusgriseus* in terms of morphological characteristics, including the humeral angle and male genitalia. The Japanese specimen was identified as *P.griseus* using a key for the East Asian species of *Picromerus* (Zhao et al. 2013), based on its morphological characteristics. However, the colouration of the connexivum of the Japanese specimen (yellow and black) did not match the above-mentioned key (entirely black). To the best of our knowledge, Japanese populations of *P.bidens* and *P.lewisi* show a high degree of intraspecific variation in the colouration of the connexivum (yellow and black to entirely black) (Figs 1c, d, 7). In conclusion, we did not use the colouration of the connexivum as a diagnostic characteristic of *Picromerus* and identified the Japanese specimen as *P.griseus*, based on the shape of the humeral angle and male genitalia.

## Checklists

### Checklist of the species of *Picromerus* occurring in Japan

#### Picromerus bidens

(Linnaeus, 1758)

446F3361-8AD0-525D-9221-AEDDD875210B

##### Distribution

Afghanistan, Albania, Algeria, Armenia, Austria, Azerbaijan, Belgium, Bosnia Herzegovina, Bulgaria, Byelorussia, Canada, China, Croatia, Czech Republic, Denmark, Estonia, Finland, France, Georgia, Germany, Greece, Hungary, Iran, Ireland, Italy, Japan (Kunashiri Island, Hokkaido, Rishiri Island, Honshu), Kazakhstan, Kirgizia, Korea, Latvia, Liechtenstein, Lithuania, Luxemburg, Macedonia, Moldavia, Mongolia, Montenegro, Netherlands, Norway, Poland, Portugal, Romania, Russia, Serbia, Slovakia, Slovenia, Spain, Sweden, Switzerland, Tajikistan, Turkey, Ukraine, United Kingdom, USA, Uzbekistan (Thomas 1994, Kanyukova and Marusik 2006, Rider 2006, Takai and Ishikawa 2012, Aukema et al. 2013, Zhao et al. 2013, Ishikawa 2016, Roca-Cusachs et al. 2020).

#### Picromerus griseus

(Dallas, 1851)

193F1E3C-A7F4-5123-9FA6-6231EDC725B0

http://en.wikipedia.org/wiki/Atypus_affinis

##### Distribution

Bangladesh, Bhutan, China, Indonesia, Japan (Ishigaki Island), Myanmar, Pakistan, Taiwan (Thomas 1994, Rider 2006, Zhao et al. 2013, Zheng and Lin 2013, present study).

#### Picromerus lewisi

Scott, 1874

2C14B9D6-627C-5C5A-9583-5EBFCA81B192

##### Distribution

China, Japan (Hokkaido, Honshu, Sado Island, Awa Island, Shikoku, Kyushu, Tsushima Island, Shimokoshiki Island, Shimoshima Island), Kazakhstan, Korea, Russia, Taiwan (Yasunaga et al. 1993, Thomas 1994, Rider 2006, Takai and Ishikawa 2012, Aukema et al. 2013, Zhao et al. 2013, Zheng and Lin 2013, Ishikawa 2016, Nozaki et al. 2016, Roca-Cusachs et al. 2020).

## Identification Keys

### Key to the species of *Picromerus* occurring in Japan

**Table:** 

1	Humeral angle of pronotum posteriorly with a distinct subapical prominence (Figs 1a, 2a, b); lateral sides of abdomen without black spots on ventral surface; known from Ryukyu Islands	*Picromerusgriseus* (Dallas, 1851)
–	Humeral angle of pronotum without distinct subapical prominence (Figs 1c, d, 2b, c); lateral sides of abdomen with black spots on ventral surface; known from Japan proper and its surrounding islands	2
2	Humeral angle of pronotum strongly protruding laterad, acute at apex (Fig. 2b); posterior margin of genital capsule weakly curved inwards in middle part (Fig. 4b); paramere weakly curved inwards in apical part in dorsal and caudal views, distinctly concave in inner margin in dorsolateral view (Figs 3b, 5b, 6b)	*Picromerusbidens* (Linnaeus, 1758)
–	Humeral angle of pronotum weakly protruding laterad, obtuse at apex (Fig. 2c); posterior margin of genital capsule strongly curved inwards in middle part (Fig. 4c); paramere strongly curved inwards in apical part in dorsal and caudal views, slightly concave in inner margin in dorsolateral view (Figs 3c, 5c, 6c)	*Picromeruslewisi* Scott, 1874

The key below is based on morphological characteristics and colouration that are stable in the Japanese populations and the distribution of three species in Japan.

## Supplementary Material

XML Treatment for Picromerus griseus

XML Treatment for Picromerus bidens

XML Treatment for Picromerus griseus

XML Treatment for Picromerus lewisi

## Figures and Tables

**Figure 1a.**
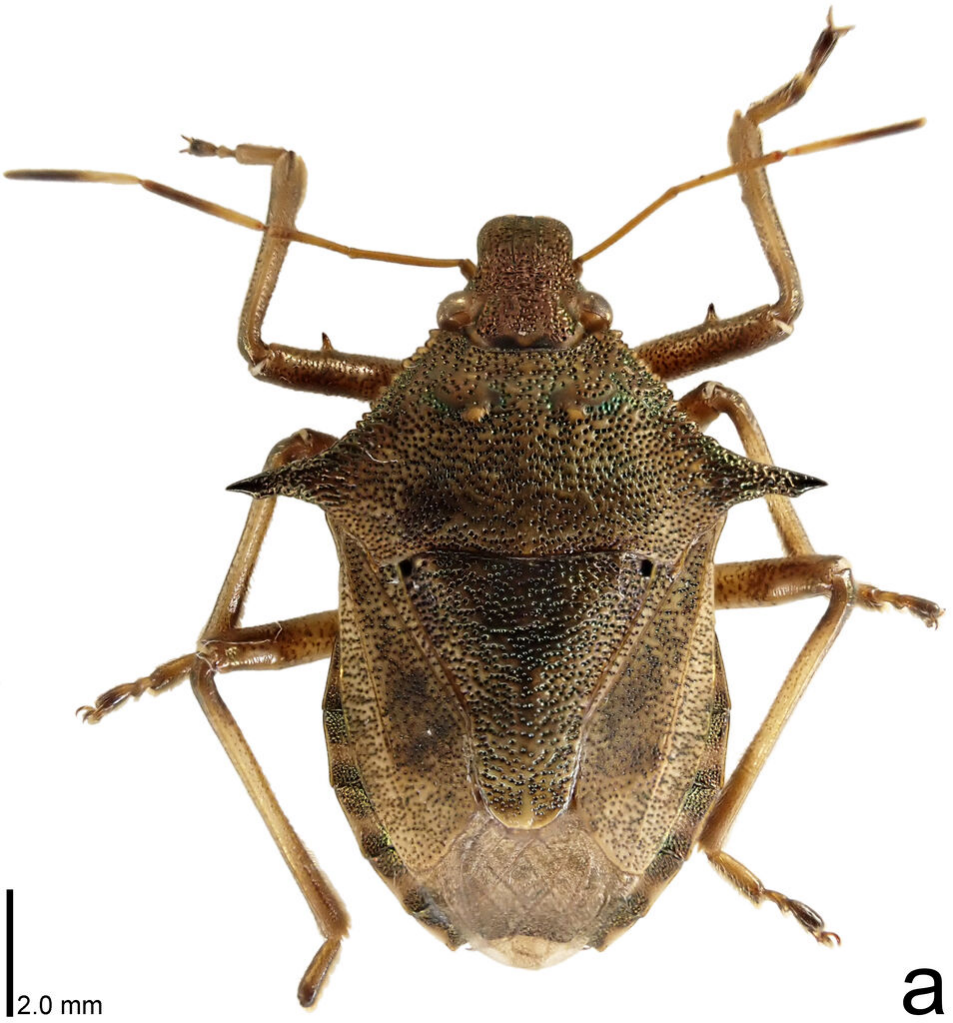
*Picromerusgriseus*, dorsal view.

**Figure 1b.**
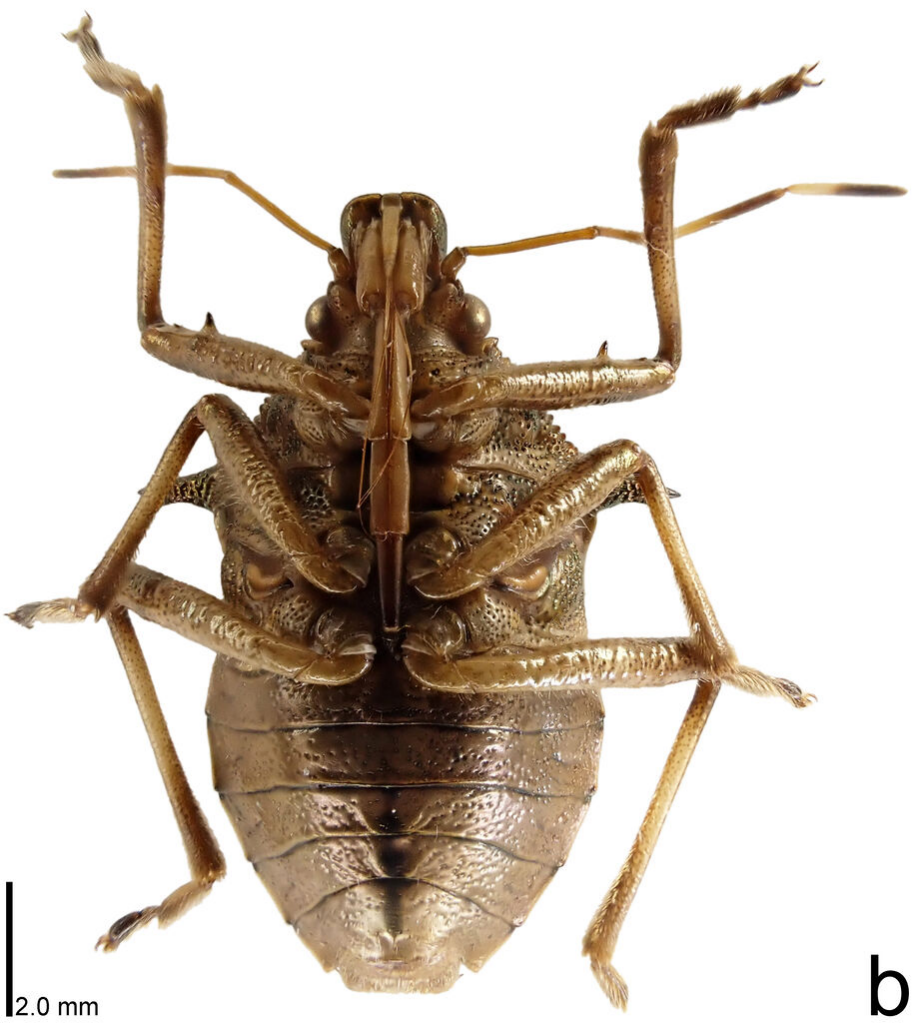
*Picromerusgriseus*, ventral view.

**Figure 1c.**
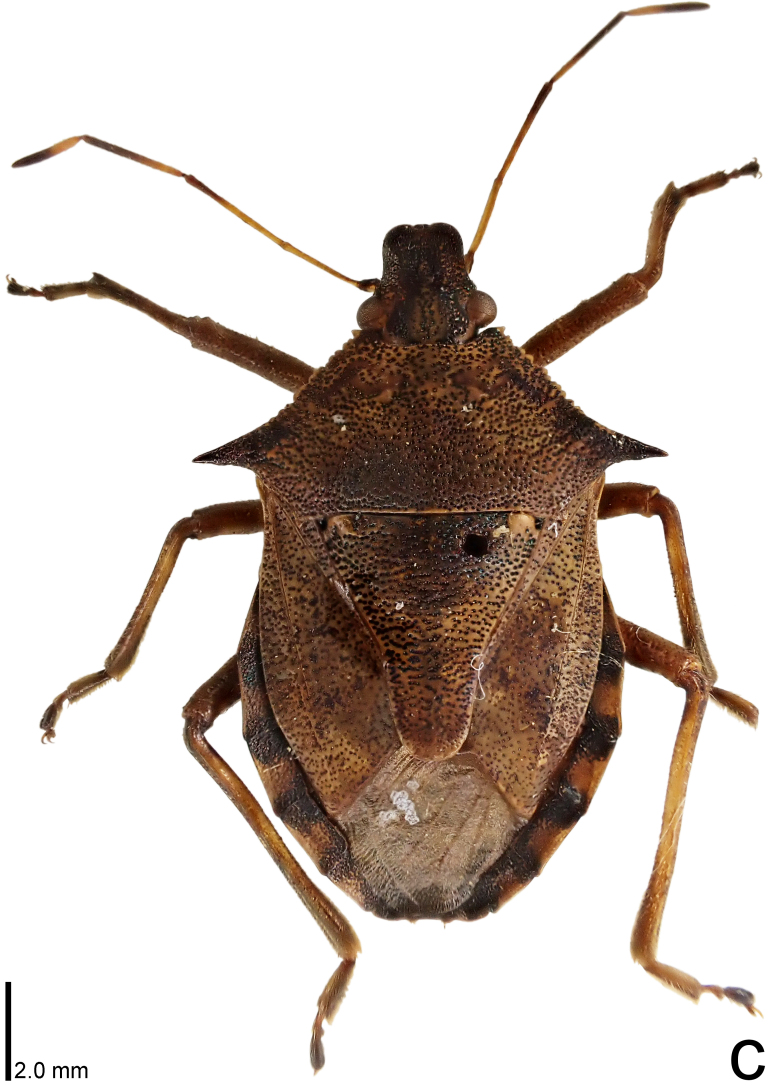
*Picromerusbidens*, dorsal view.

**Figure 1d.**
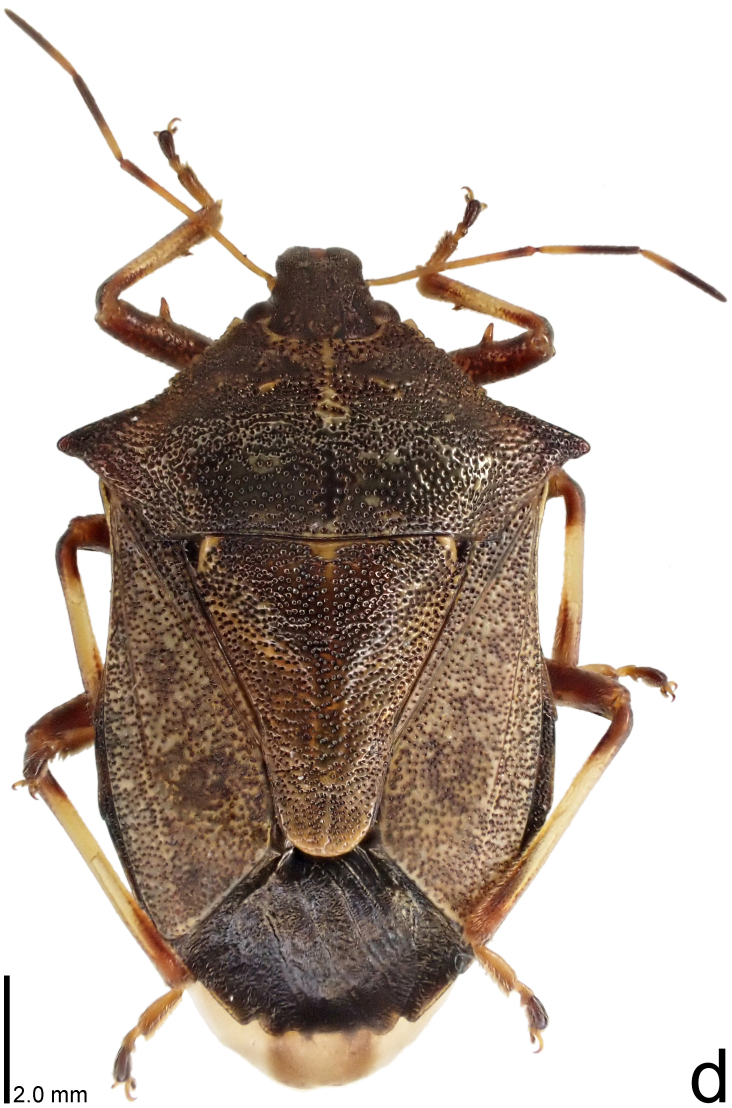
*Picromeruslewisi*, dorsal view.

**Figure 2a.**
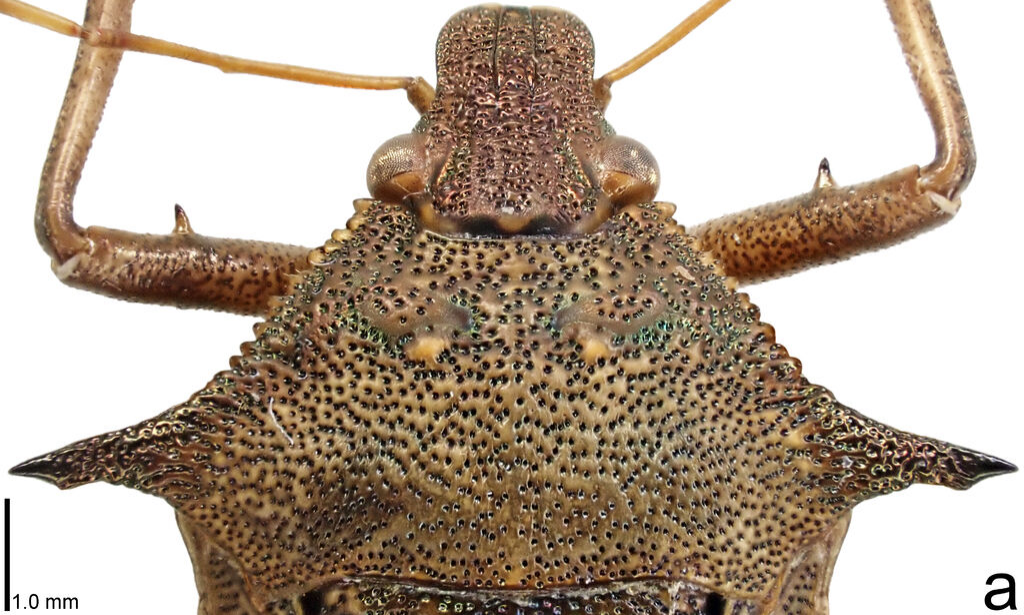
*Picromerusgriseus*.

**Figure 2b.**
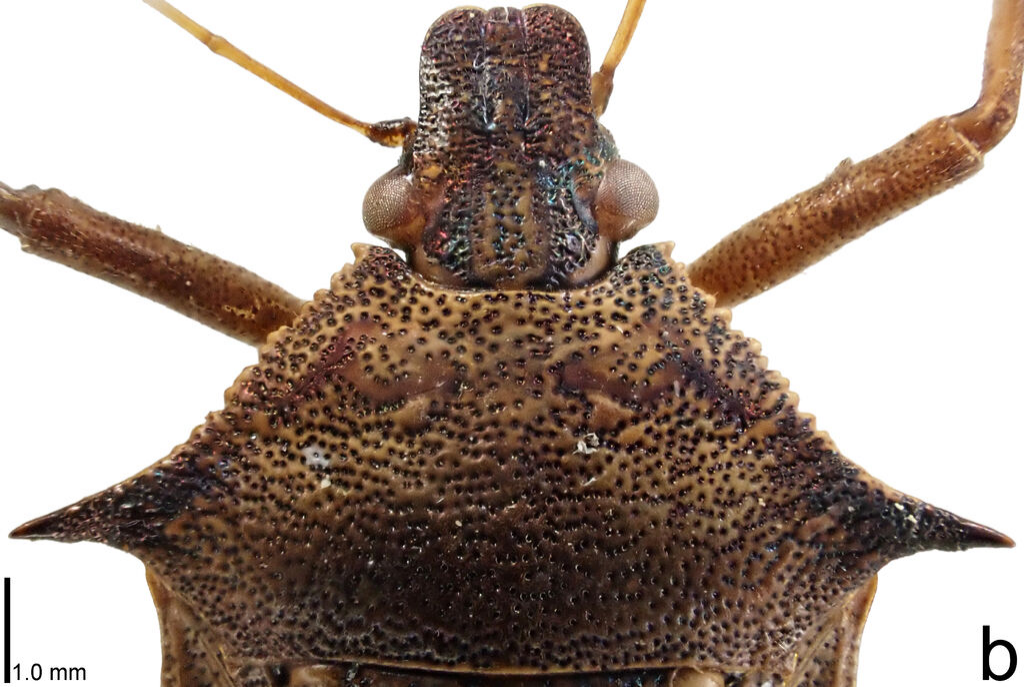
*Picromerusbidens*.

**Figure 2c.**
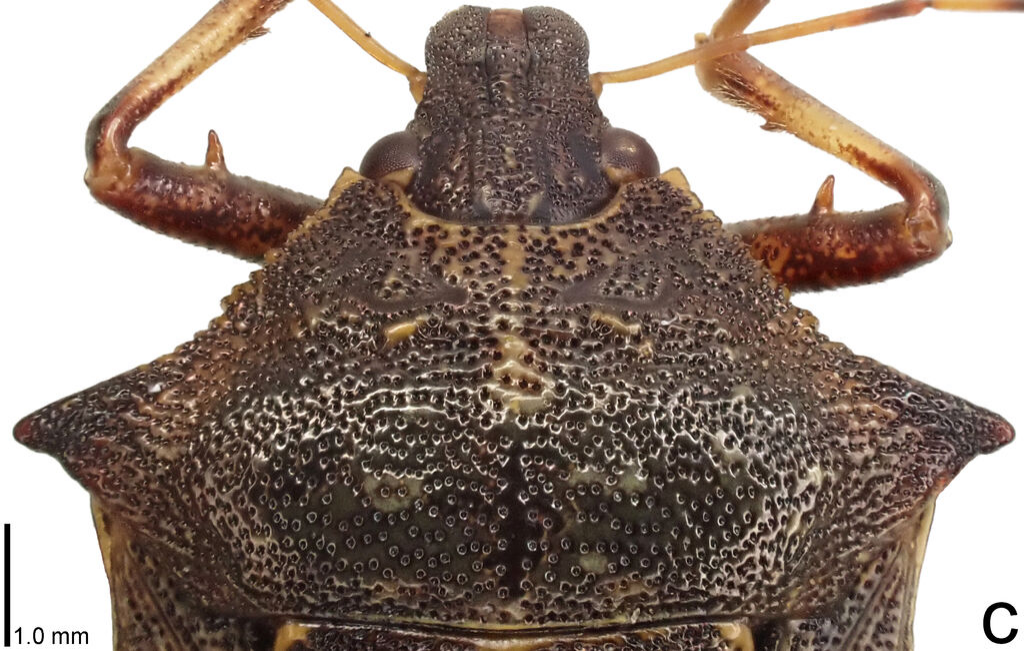
*Picromeruslewisi*.

**Figure 3a.**
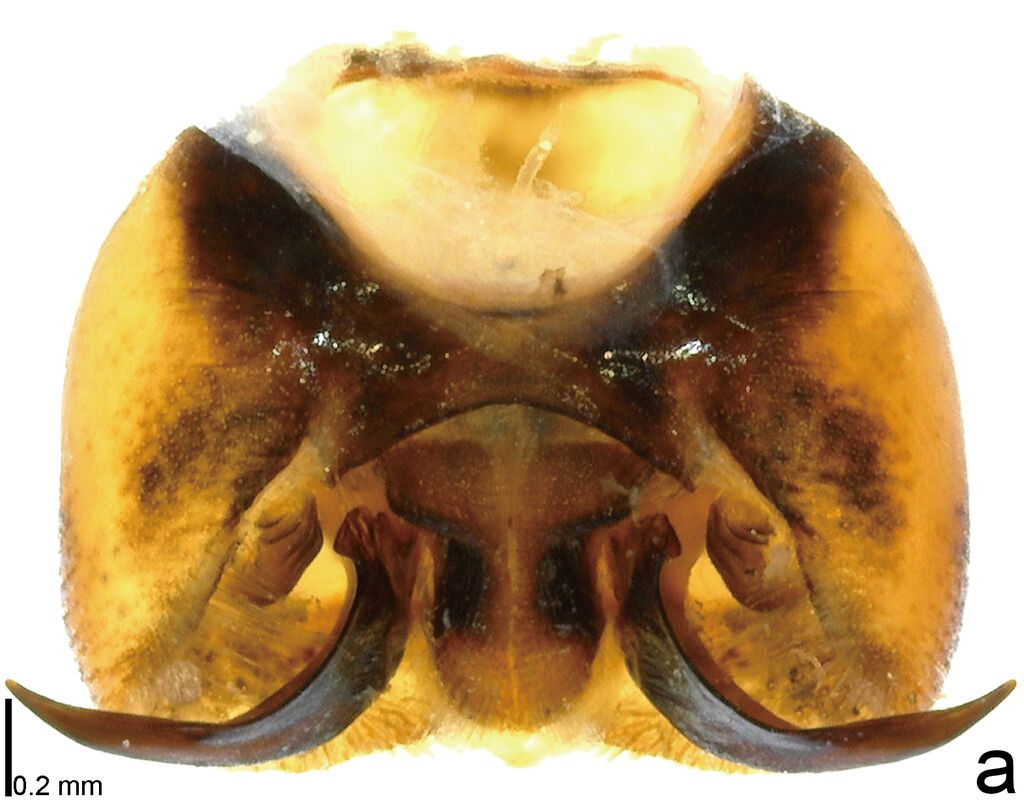
*Picromerusgriseus*.

**Figure 3b.**
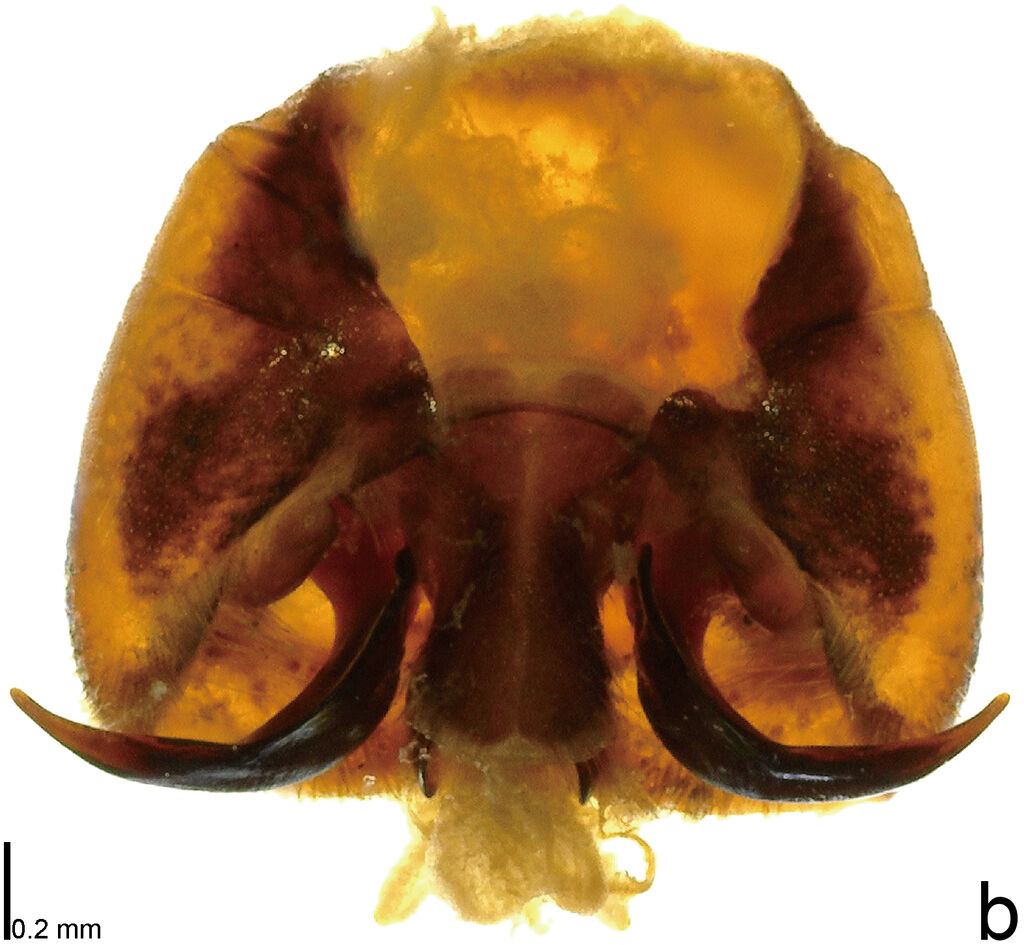
*Picromerusbidens*.

**Figure 3c.**
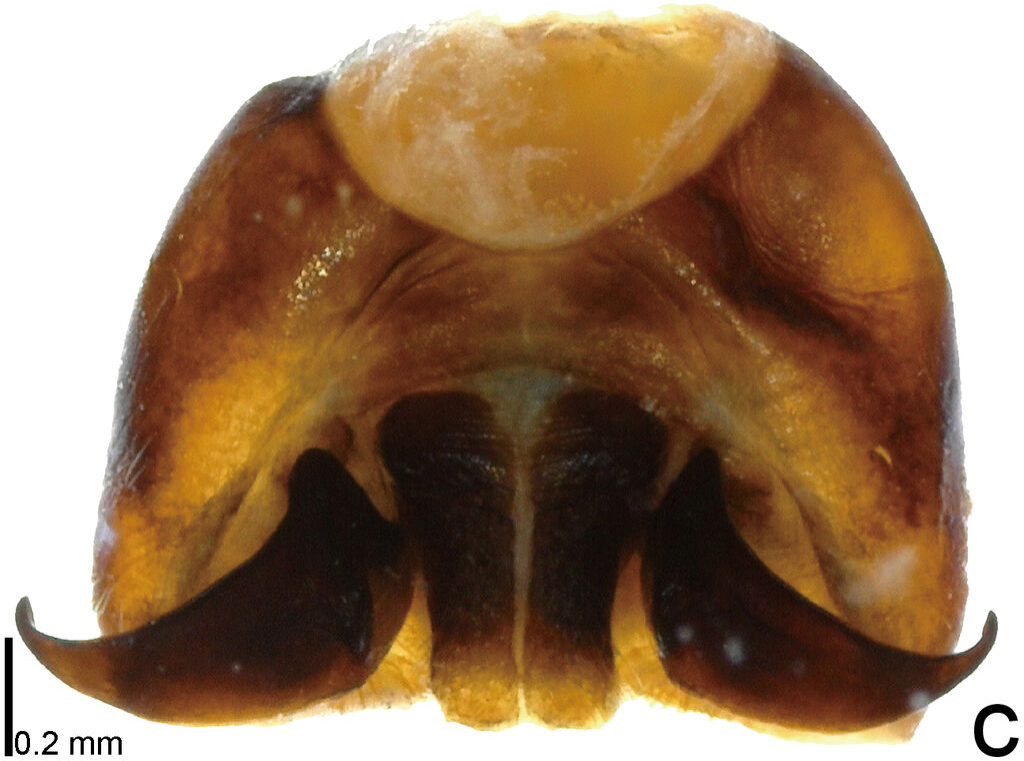
*Picromeruslewisi*.

**Figure 4a.**
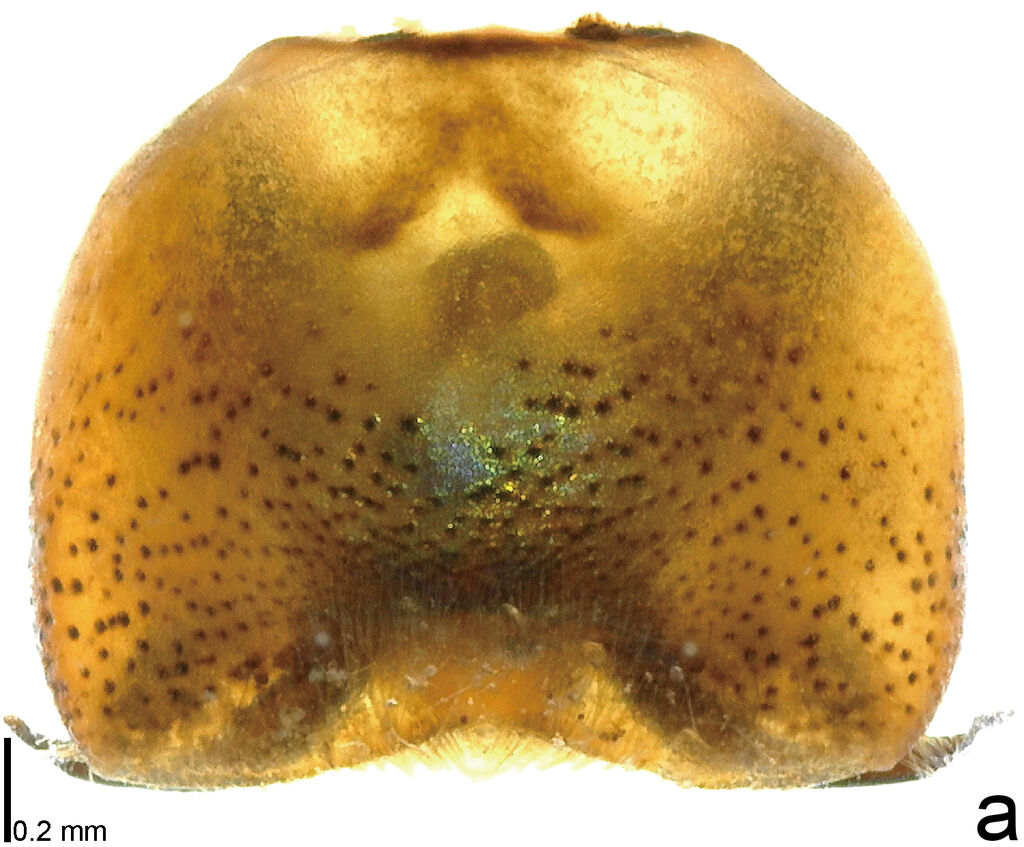
*Picromerusgriseus*.

**Figure 4b.**
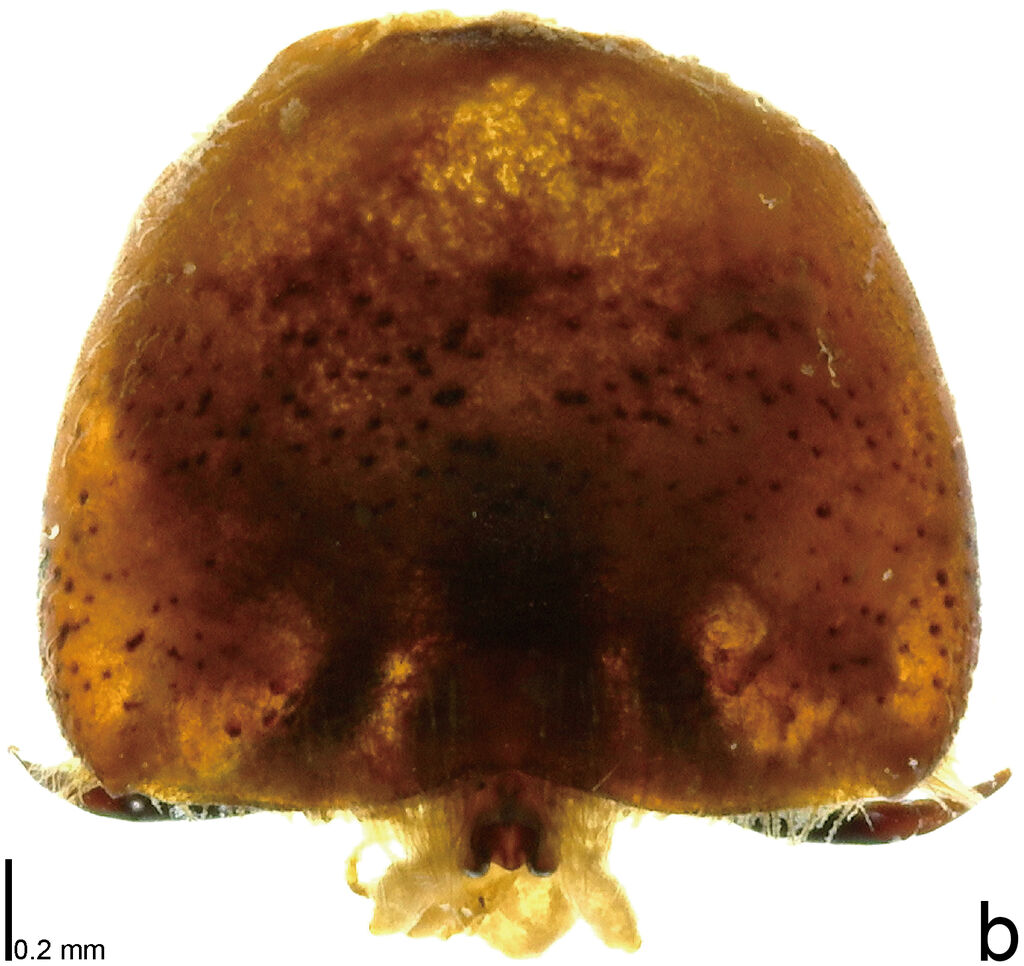
*Picromerusbidens*.

**Figure 4c.**
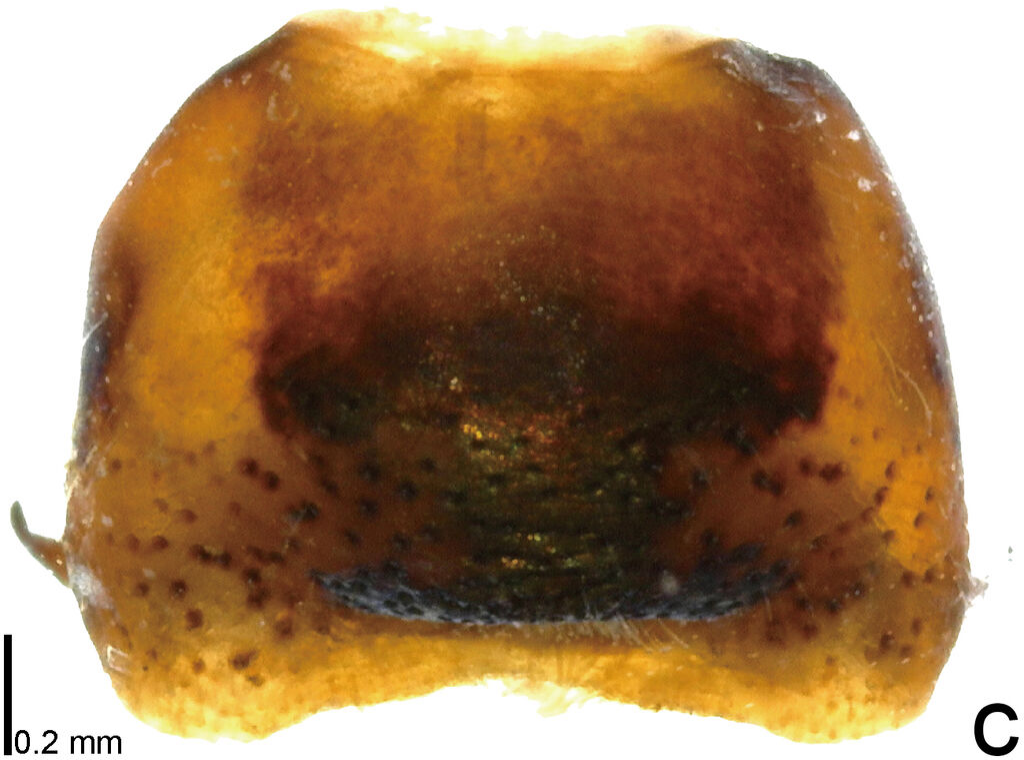
*Picromeruslewisi*.

**Figure 5a.**
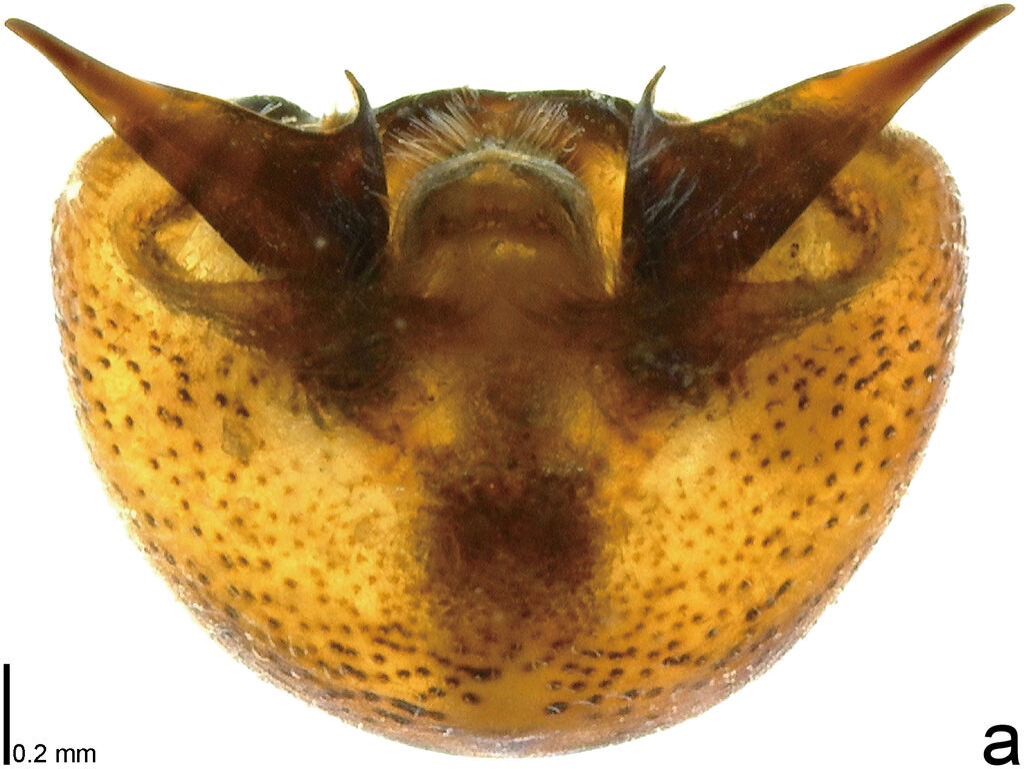
*Picromerusgriseus*.

**Figure 5b.**
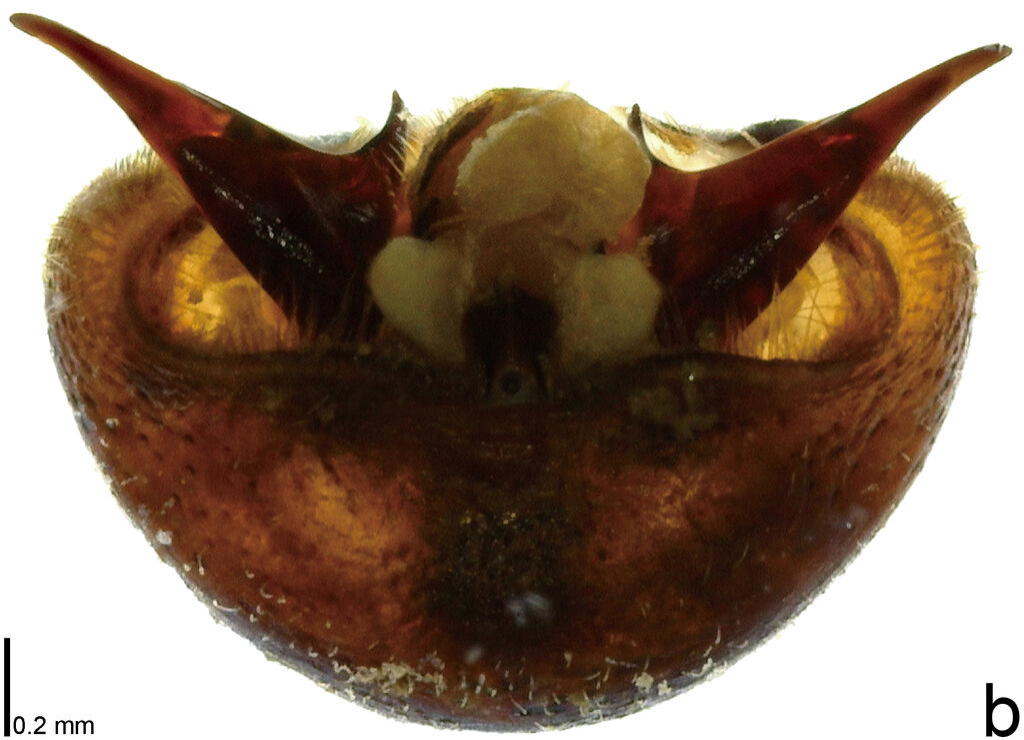
*Picromerusbidens*.

**Figure 5c.**
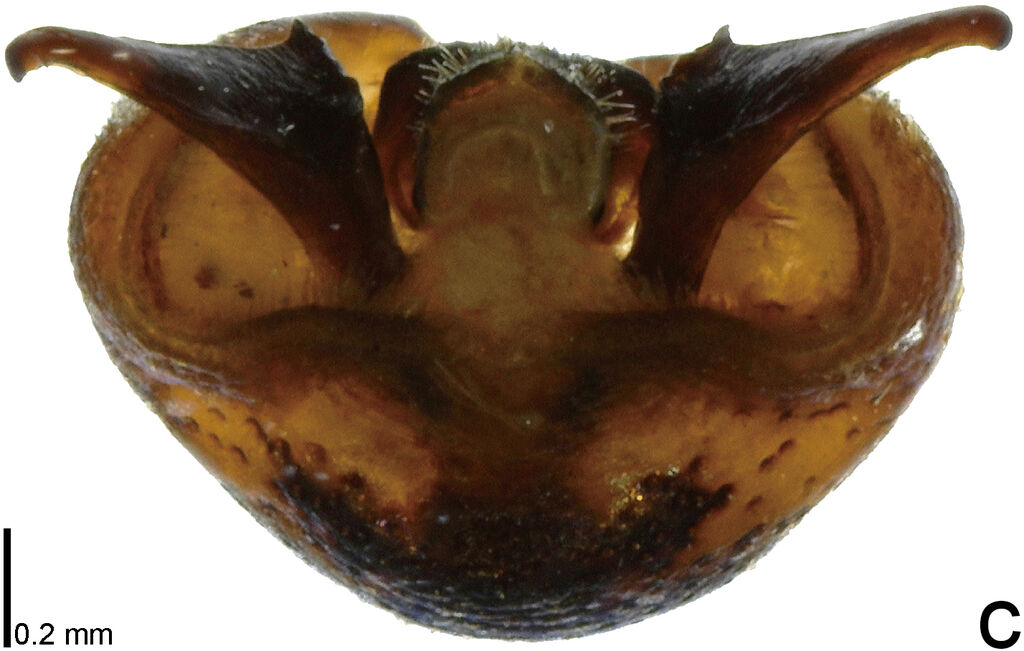
*Picromeruslewisi*.

**Figure 6a.**
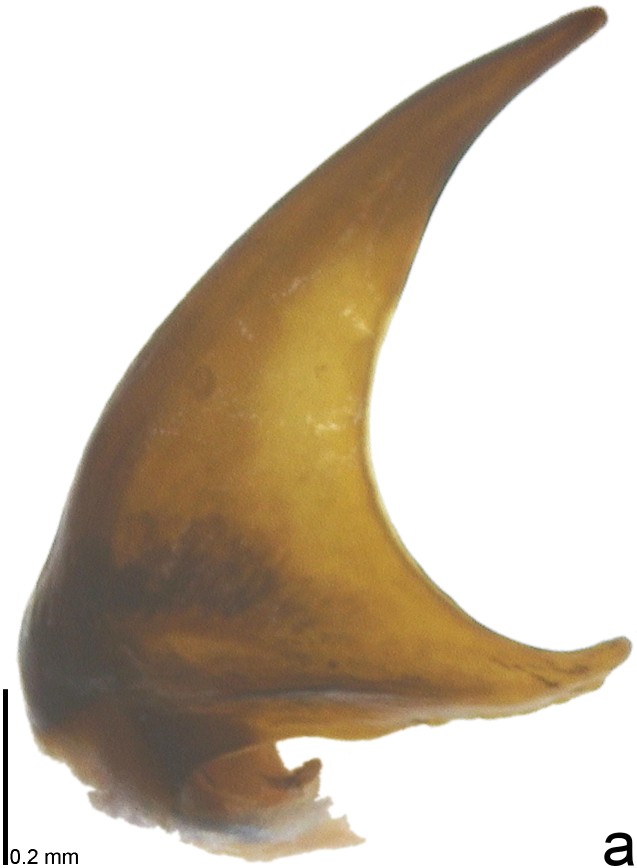
*Picromerusgriseus*.

**Figure 6b.**
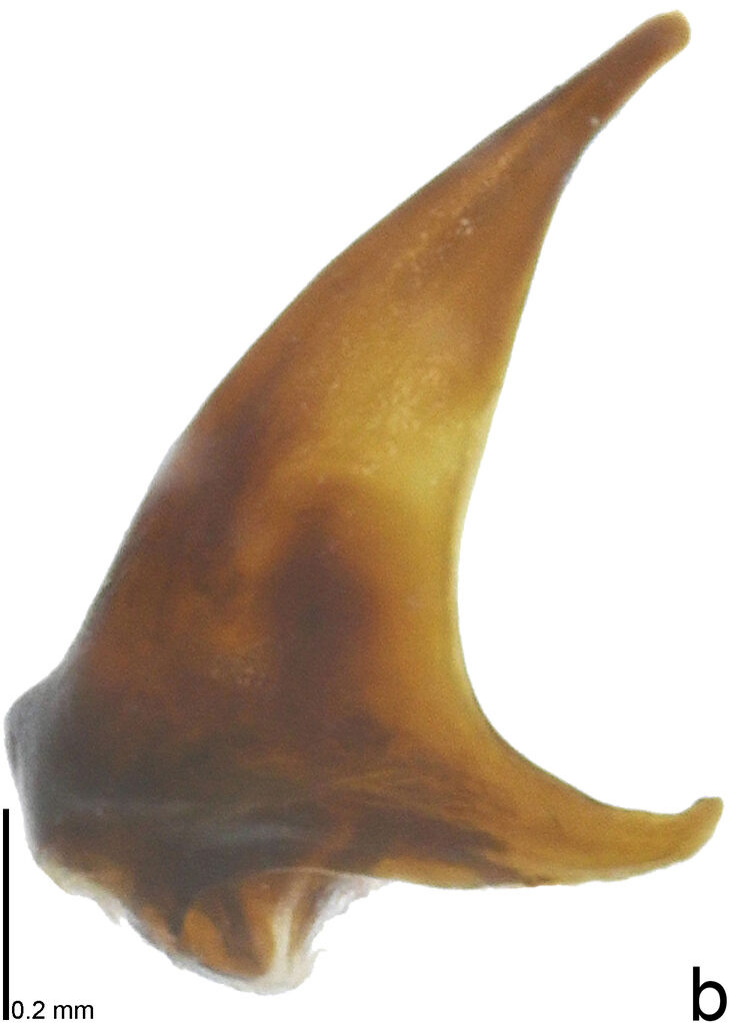
*Picromerusbidens*.

**Figure 6c.**
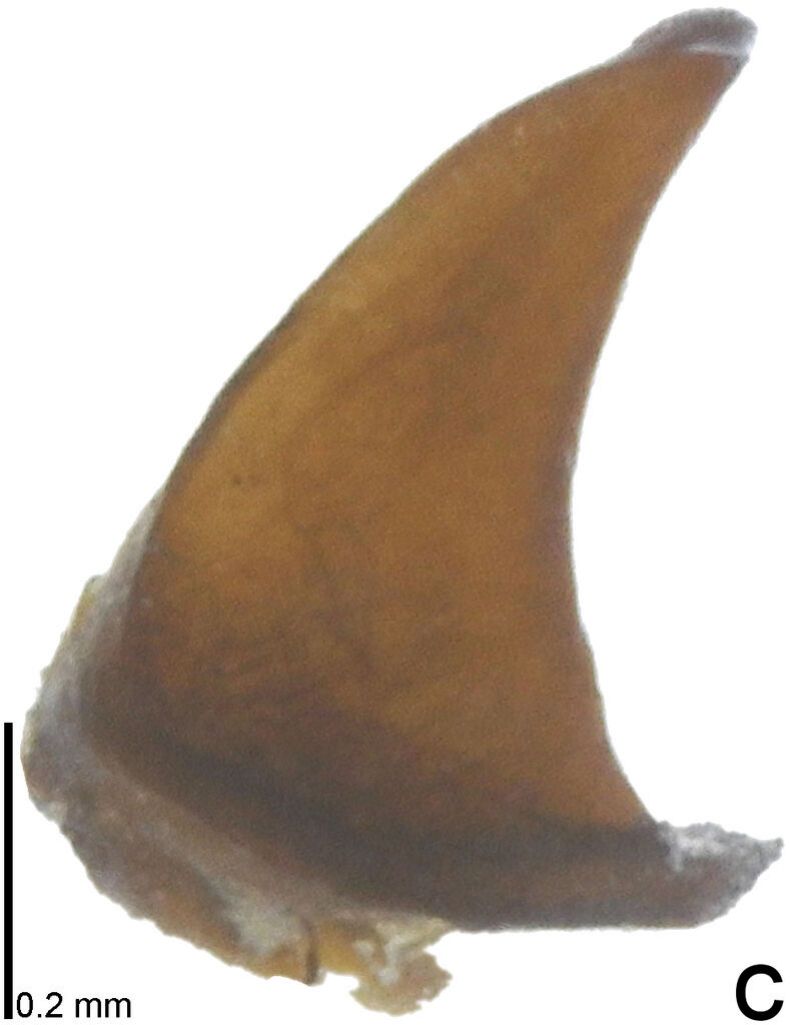
*Picromeruslewisi*.

**Figure 7a.**
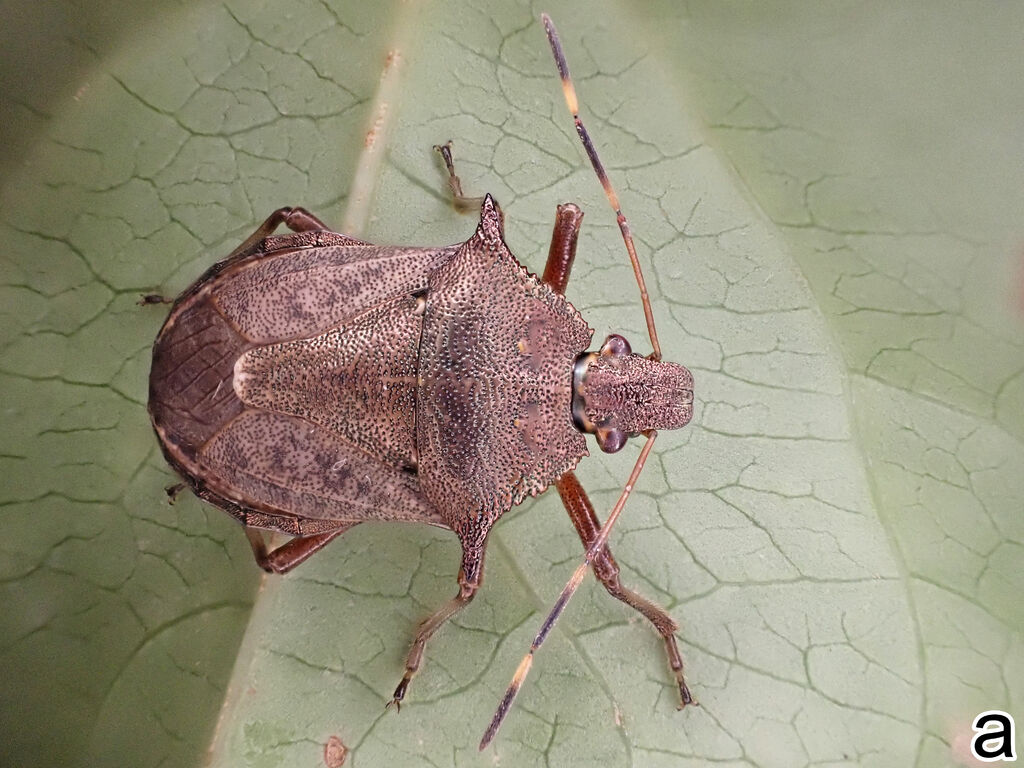
*Picromerusbidens*.

**Figure 7b.**
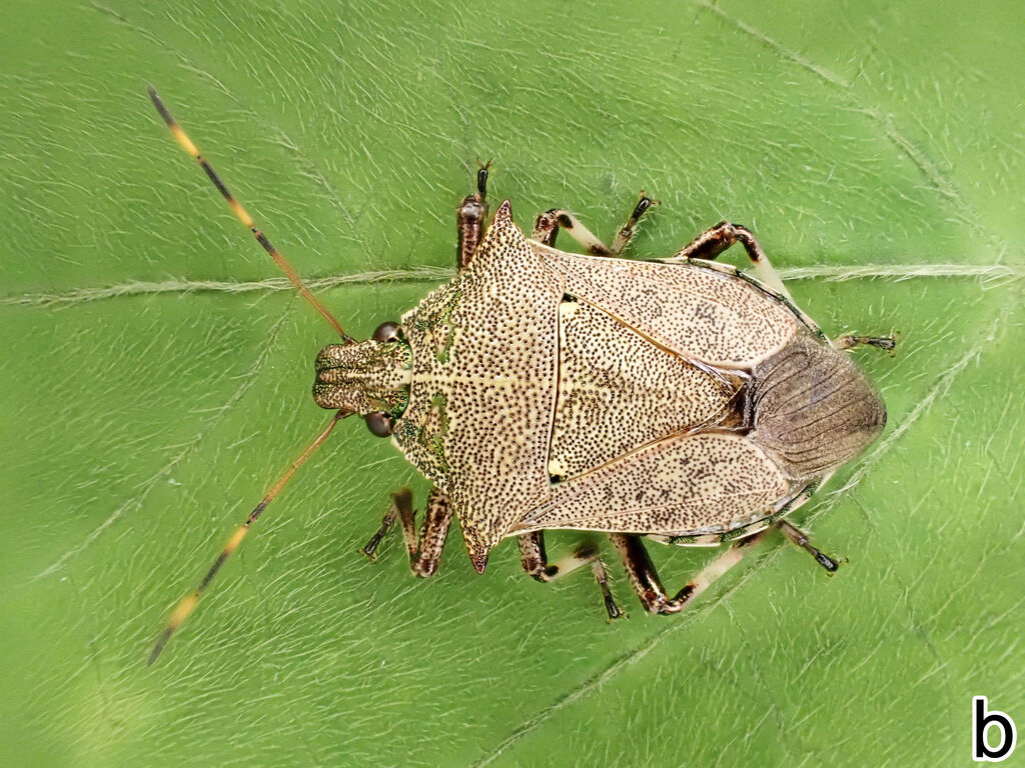
*Picromeruslewisi*.
